# The effects of perinatal bisphenol A exposure on thyroid hormone homeostasis and glucose metabolism in the prefrontal cortex and hippocampus of rats

**DOI:** 10.1002/brb3.1225

**Published:** 2019-02-13

**Authors:** Xiaobin Xu, Shijun Fan, Yuanqiao Guo, Ruei Tan, Junyu Zhang, Wenhua Zhang, Bing‐Xing Pan, Nobumasa Kato

**Affiliations:** ^1^ Laboratory of Fear and Anxiety Disorders, Institute of Life Science Nanchang University Nanchang China; ^2^ School of Statistics University of International Business and Economics Beijing China; ^3^ Tan Clinic Tokyo Kanagawa Japan; ^4^ Medical Institute of Developmental Disorders Research Showa University Tokyo Japan

**Keywords:** behavioral deficits, bisphenol A, deiodinase enzyme, glucose metabolism, thyroid hormone homeostasis, thyroid hormone transporter

## Abstract

**Introduction:**

Bisphenol A (BPA) is an endocrine disruptor widely used to manufacture consumer goods. Although the thyroid hormone (TH) disrupting potential of BPA has been thought to be responsible for the neuropsychiatric deficits in the animals that experienced perinatal BPA exposure, the TH availability change at the level of specific brain structures has not been subject to systematic investigation.

**Methods:**

In the present study the impacts of perinatal BPA exposure (0.1 mg/L in drinking water) spanning gestation and lactation on TH homeostasis in the prefrontal cortex (PFC) and hippocampus were assessed in male Sprague–Dawley rats at postnatal day 21 (PND21) and PND90. As TH regulates brain glucose metabolism at multiple levels，the effects of BPA treatment on glucose metabolism in the brain tissues were also assessed in adult rats.

**Results:**

The results showed heterogeneous changes in TH concentration induced by BPA between serum and brain tissues, additionally, in the BPA–treated pups, up–regulated expression of the TH transporter monocarboxylate 8 mRNA at PND21 and increased type 3 iodothyronine deiodinase mRNA expressions at PND21 and PND90 were observed. Meanwhile, decreased glucose metabolism was seen in the PFC and hippocampus, while deficits in locomotor activity, spatial memory and social behaviors occurred in BPA‐treated groups.

**Conclusion:**

These data support the concept that the developing brain possesses potent mechanisms to compensate for a small reduction in serum TH, such as serum hypothyrodism induced by BPA exposure, however, the long‐term negative effect of BPA treatment on TH homeostasis and glucose metabolism may be attributable to neuropsychiatric deficits after mature.

## 1 INTRODUCTION

Bisphenol A (BPA) is an ubiquitous xenoestrogen and a raw material widely used in the manufacturing of a variety of consumer goods and is easily ingested by humans (Brede, Fjeldal, Skjevrak, & Herikstad, 2003). As a result, about 90% of the population has detectable levels of BPA in their urine, since children typically have higher level than adult (Calafat, Ye, Wong, Reidy, & Needham, 2008; Edginton & Ritter, 2009; Li et al., 2017).

BPA is completely and rapidly absorbed from the gastrointestinal tract (Volkel, Colnot, Csanady, Filser, & Dekant, 2002). Our previous study has demonstrated that perinatal constant BPA exposure at a low dose of 0.1 mg/L via maternal drinking water, which is equivalent to about 15 μg kg^‐1^ day^‐1^ BPA intake (Xu et al., 2012), induced a mean BPA level of 1.7 ng/ml in serum in 21‐day‐old pup rats which is lower than the mean value observed in girls and boys (Chen et al., 2015; Komarowska et al., 2015), and caused hyperactivity, impaired spatial memory and deficit of contextual fear learning in adulthood (Xu et al., 2017, 2014). These evidences suggest that the serum BPA level in children is sufficient to lead to a spectrum of adverse consequences on brain development and behavior in rodents ranging from the hypotrophy of dendritic spine in the developing neurons to neuropsychiatric deficits (Xu et al., 2007, 2014, 2013). Concerning the relevance of extrapolating animal data to human risk assessment, a lot of researches have shown that developmental exposure to very low doses of BPA (that produce blood levels in animals below those in humans) all relate to disease trends in humans (Talsness, Andrade, Kuriyama, Taylor, & Saal, 2009), strongly suggesting the validity of rodent models for human risk assessment of BPA. Although the mechanisms underlying the actions of BPA on the development of brain are not well understood, one hypothesis was advanced that affected thyroid hormone (TH) homeostasis may be involved in the adverse consequences of BPA exposure, because of the change in serum TH level in newborns perinatally exposed to BPA (Birnbaum et al., 2012; Xu et al., 2007; Zoeller, Bansal, & Parris, 2005) and profound actions of TH on brain development and function (Ahmed, El‐Gareib, El‐Bakry, Abd El‐Tawab, & Ahmed, 2008).

For acting in the brain, firstly serum TH has to enter the brain which does not take place by passive diffusion but requires the involvement of specific transporters that enable the passage of TH across blood–brain barriers (BBB) (Visser, Friesema, & Visser, 2011). Of the several proteins capable of TH transport, present in the rodent brain (Kinne, Schülein, & Krause, 2011), monocarboxylate transporter 8 (MCT8) and organic anion–transporting polypeptide 1c1 (OATP1c1) are indispensable for TH entry into the brain (Ceballos et al., 2009; Karapanou & Papadimitriou, 2011; Mayerl et al., 2014; Mayerl, Visser, Darras, Horn, & Heuer, 2012; Tohyama, Kusuhara, & Sugiyama, 2004; Trajkovic et al., 2007). After passage into the brain thyroxine (T_4_), must be converted to triiodothyronine (T_3_) in order to initiate TH action. The type 2 iodothyronine deiodinase (DIO2), expressed in astrocytes, tanycytes and in some sensory neurons, is responsible for converting T_4_ to T_3_ (Guadaño‐Ferraz, Escámez, Rausell, & Bernal, 1999; Guadaño‐Ferraz, Obregón, St Germain, & Bernal, 1997) in the brain. Meanwhile, inactivation of T_3_ can also take place within the brain and is catalyzed by the neuronally localized DIO3 (Tu et al., 1999). Because of the complexities of transmembrane TH transport and TH metabolism in the brain, the serum TH level may be not an accurate index of the functional concentrations in the brain. In fact, discrepancy between plasma and tissue TH homeostasis has been reported in genetic diseases such as MCT8 deficiency (Dumitrescu & Refetoff, 2013), as well as in some patients subjected to replacement therapy after thyroidectomy who still display symptoms related to hypothyroidism in spite of a normal plasma TH profile (Biondi & Wartofsky, 2012; Wiersinga, 2014). So far the available data leave it virtually unknown how TH availability changes at the level of specific brain tissues under BPA exposure, indeed, to clear up this problem is the precondition for investigating the role of the TH pathway in the actions of BPA on brain development and function.

Clinic data showed significant reduction in both neural activity and regional glucose metabolism in the brains of mild‐moderate hypothyroid patients which were restored to control levels following TH replacement therapy (Bauer et al., 2009; Constant et al., 2001), suggesting a direct link between thyroid activity and brain glucose metabolism. The present study would first provide a detailed insight into the effects of perinatal BPA exposure on brain subregion–specific TH homeostasis and glucose metabolism which would help evaluate the role of the TH pathway in BPA–induced adverse behavioral and cognitive impairment in animals.

## 2 MATERIALS AND METHODS

### 2.1 Animal care and BPA treatment

**Figure 1:**
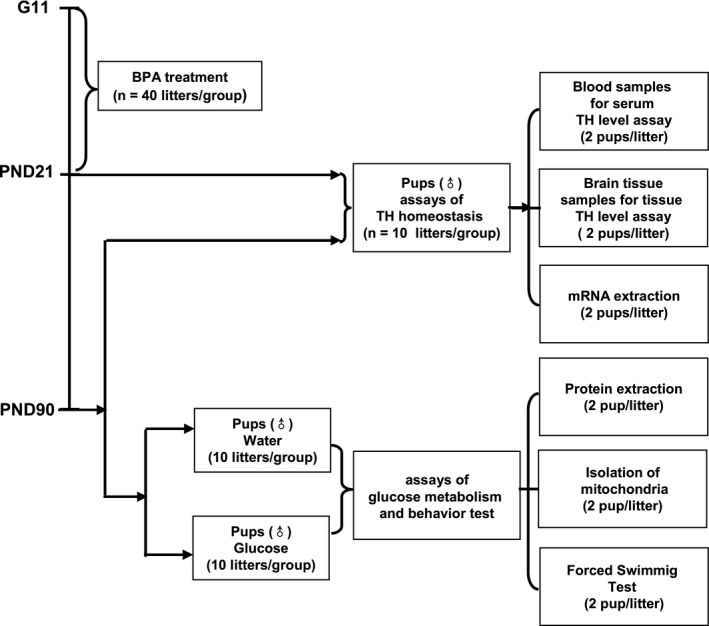
Scheme showing the time course of the experiments and the use of animals

According to our previous results, here we used a single dose of 0.1 mg/L BPA in maternal drinking water to treat the pregnant and lactational dams. Female Sprague–Dawley rats (body weight: 250–275 g) at gestational day (G) 11 were housed individually in metal cages and maintained on a 12‐hr light, 12‐hr dark lighting regimen. The ambient room temperature was 18°C. Glass drinking bottles were used during this research. All rats were watered with glass bottles. Dams were divided into two groups and administered with water or BPA (2,2‐bis(4‐hydroxyphenol) propane) (Aldrich Chemical Company, Canada) dissolved in 0.01% ethanol in drinking water (unchlorinated purified water) at 0.1 mg/L from G11 to lactation day (L) 21. Food intake, water consumption and body weight of the dams were recorded every day throughout the experiment period (G11 to L21). The offspring were housed by sex (*n* = 3–4/cage) from postnatal day (PND) 21 and given tap water till 90 days of age. The dams (*n* = 120/group) were raised in three batches for three independent experiments. The female sex hormone estrogen has a well–known indirect effect on thyroid economy, such as increasing the T_4_ binding globulin, while the direct effects of estrogen on thyroid cells have been described more recently (Santin & Furlanetto, 2011). In addition, numerous studies have reported that estrogens and their receptors have multiple implications for glucose homeostasis (Mauvais‐Jarvis, Clegg, & Hevener, 2013), and that the menstrual cycle significantly affects glucose control in female animals (Barata, Adan, Netto, & Ramalho, 2013; Diamond, Simonson, & DeFronzo, 1989). With these considerations, in the present study we used only male pups to avoid the uncertainty of the data in female rats. The time course and the use of animals in each independent experiment are shown in Figure 1. All animal procedures were in agreement with the general guidelines of animal care and the recommendations of the Medical Ethics Committee of Nanchang University.

### 2.2 Measurement of circulating and tissue THs

The dams (at G21) and male pup rats subjected (at PND21 and PND90, respectively) were killed by decapitation after anesthesia, and the whole blood was collected; then the brains were immediately removed from the skull, rinsed in saline to remove residual blood, the prefrontal cortex (PFC) and hippocampus of both hemispheres were quickly removed from the brains and quickly frozen in liquid nitrogen and stored at −80°C until usage. Once the blood was collected, it was immediately centrifuged at 5,000 *g* for 10 min, the serum was used for ELISA assay of the circulating total T_4_ (BioVision), total T_3_ (BioVision), free T_4_ (CUSABIO) and free T_3_ (Bayer Medical Ltd). Brain tissue total T_4_ and total T_3_ assays were performed using High performance liquid chromatography tandem mass spectrometry (HPLC‐MS/MS). Briefly, for assaying the TH level in the PFC and hippocampus of the PND90 rats, we added 200 mg of each tissue sample into 1 ml of 85:15 (v/v) acetonitrile/0.1 mol/L HCl solution containing internal standards, which were then extracted in an ultrasound bath (Scientz‐IID, Scientz, China) for 25 min and homogenized through a grinder (Precellys 24, Bertin Technologies, France) using three homogenization steps of 45 s with 60 s pause at 5,000 rpm. After transferring the homogenate into a centrifugal tube and diluting it to 2 ml with acetonitrile, the solution was left in the above mentioned ultrasound bath for another 15 min and then centrifuged for 15 min at 1,300 *g* at room temperature. The supernatant was placed in a new glass centrifuge tube and was subjected to liquid/liquid extraction with 1 ml hexane for three times. After every extraction the upper phase (hexane) was discarded and the lower phase (acetonitrile) was dried under a stream of nitrogen at 45°C; the dried residue was submitted for derivatization (Donzelli et al., 2016). Derivatization and HPLC‐MS/MS analysis were carried out using a quaternary HPLC pump (WATERS Xevo TQ MS ACQUITY UPLC System, WATERS, USA). The binary gradient system consisted of 5% acetonitrile in water containing 0.1% of acetic acid (eluent A) and 95% acetonitrile in water containing 0.1% of acetic acid (eluent B). Gradient elution was performed according to the following elution program: 0–2.5 min, 90% A, 10% B; 2.5–8.5 min, 60% A, 40% B; 8.5–11 min, 60% A, 40% B; 11–12 min, 90% A, 10% B. The flow rate was 0.2 ml/min. The temperature of the Waters column was kept at 60°C. the HPLC‐MS/MS instrument was operated with a capillary voltage of 3.2 kV, source temperature 150°C, desolvation temperature 450°C, cone gas 55 L/h, desolvation gas 1,200 L/h (Ackermans, Kettelarij‐Haas, Boelen, & Endert, 2012). Quality control data were determined for both extraction procedures. Briefly, accuracy was defined as the ratio concentrations of T3 (0.2 and 1 ng) and T4 (1 and 10 ng); precision was defined as the coefficient of variation (standard deviation/mean) of repeated measurements within the same assay under the same conditions as described above; recovery was defined as the ratio of internal standard spiked before extraction to internal standard spiked after extraction; matrix effect was defined as the ratio of internal standard spiked after extraction to internal standard dissolved in the reconstitution solvent (Donzelli et al., 2016).

### 2.3 Quantitative real–time PCR

The male pup rats subjected (at PND21 and PND90) were killed by decapitation after anesthesia and the brains were immediately removed from the skulls, rinsed in saline to remove residual blood, the PFC and hippocampus tissues of both hemispheres were quickly extracted from the brains and quickly frozen in liquid nitrogen and stored at −80°C until usage. The total RNAs were extracted by using Trizol reagent and the samples containing 1 μg of total RNA were reverse transcribed using High Capacity RNA‐to cDNA kit (Applied Biosystems) with reference to the manufacturer's instructions. Quantitative real–time PCR analyses of the cDNA samples (30 ng) were performed with an ABI Prisms 7900 HT (Applied Biosystems) following the protocols provided by the manufacturer. The following primers were used for qPCR: MCT8 forward (5′‐TGGTTACTTCGTCCCCTACG‐3′), reverse (5′‐CCAGGGATGGAGTCACTGAT‐3′); OATP1c1 forward (5′‐GCAAATGTTCAGACTCAAAATGGG‐3′), reverse (5′‐ATATAATGTTCTTTCCACTCCGGC‐3′); DIO2 forward (5′‐ACTCGGTCATTCTGCTCAAG‐3′), reverse (5′‐CAGACACAGC GTAGTCCTTC‐3′); DIO3 forward (5′‐CTCGAACTGGCAACTTTGT‐3′), reverse (5′‐GTGAGATGCTGGCGACTTAT‐3′). The fluorescent signals from specific transcripts were normalized against that of the glyceraldehyde‐3‐phosphate dehydrogenase gene and calculated as the means of threshold cycle values (△Ct) or quantified as fold changes by the 2^−△△Ct^ method. For each gene, we used the mean expression level of control rats at PND21 as the internal control (100%).

### 2.4 Biochemical analysis

#### 2.4.1 Oral glucose administration and tissue collection

Considering that anesthetics impair glucose metabolism (Park et al., 2017; Zuurbier, Keijzers, Koeman, Wezel, & Hollmann, 2008) and the uneven tolerance of anesthesia between different individuals observed in our preliminary experiment, we applied for the ethical approval for using nonanesthetized rats in biochemical analysis. At PND90, the male pup rats subjected were administered glucose (1 g/kg) or water by oral gavage. Glucose or water was given in a dose of 2 ml/kg. Two hours after glucose administration, the animals were killed by rapid decapitation. Brains were rapidly removed, and the tissues subjected were dissected from both hemispheres on ice–cold glass plates. The tissues were homogenized in five volumes of phosphate‐buffered saline and centrifuged at 20,000 *g* for 30 min at 4°C. Half of the supernatants were then deproteinized using the Deproteinizing Sample Preparation Kit (Abcam) for the estimation of of lactate and pyruvate . Briefly, 500 μl of sample homogenates were mixed with 100 μl of ice–cold TCA buffer containing 10 mM Tris‐HCL, 10% trichloroacetic acid, 25 mM NH4OAc, and 1 mM Na2EDTA (pH 8.0), incubated on ice for 15 min, and then centrifuged at 12,000 *g* for 5 min at 4°C. To the other half of supernatant protease inhibitors (diluted 1:100, Takara Bio.) were added and stored at −80°C until usage for the assays of the key enzymes mediating the glucose metabolism in the brain. The protein concentration was determined by using the Bradford reagent (Bio‐Rad).

#### 2.4.2 Lactate and pyruvate analysis

The Lactate fluorometric assay kit (Biovision Inc., USA) and Pyruvate fluorometric pyruvate assay kit (Biovision Inc., USA) were used for measurement of lactate and pyruvate according to the manufacturer's instructions. Briefly, 50‐μl of each sample was transferred to 96‐well plates in duplicate along with lactate or pyruvate standards. Then a 50 μl reaction mix buffer (46 μl lactate assay buffer or 47.6 μl pyruvate assay buffer, 2 μl lactate probes or 0.4 μl pyruvate probes, and 2 μl of enzyme mix) was added to each test sample and lactate or pyruvate standards. After incubation at room temperature for 30 min, fluorescence was measured at Ex/Em = 535/590. The lactate or pyruvate concentration in the samples was calculated from a standard curve and expressed as nmol/mg protein.

#### 2.4.3 ELISA for hexokinase, phosphofructokinase and pyruvate kinase

Hexokinase, phosphofructokinase and pyruvate kinase in the brain tissues were determined using ELISA kits (ElAab Science Co., Ltd., China; and USCN Life Science Inc., China respectively) according to the manufacturer's instructions. Briefly, the tissues were homogenized in five volumes of phosphate‐buffered saline and centrifuged at 20,000 *g* for 30 min at 4°C. To the supernatant protease inhibitors (diluted 1:100, Takara Bio.) were added and stored at −80°C until usage for the assays of the key enzymes mediating the glucose metabolism in the brain. For the analysis of the kinases, 100 μl of the supernatant of each sample was transferred to precoated 96‐well ELISA plates in duplicate along with hexokinase standards, phosphofructokinase standards and pyruvate kinase standards respectively. The absorbance at a wavelength of 450 nm was measured using iMark^TM^ Imcroplate Reader (Bio‐Rad). The concentration of the three enzymes in samples was calculated from a standard curve and expressed as ng/mg protein (for hexokinase and phosphofructokinase) or Units/mg protein (for pyruvate kinase) respectively.

#### 2.4.4 Isolation of mitochondria

Isolation of the crude mitochondrial fraction from the brain tissues was performed according to the procedure described by Wernicke et al. (2010). Briefly, to 200 mg of each sample four volumes of homogenization buffer (containing 5 mol/L HEPES/NaOH, pH 7.4, 300 mmol/L sucrose and 1 mmol/L Na^+^/EDTA with the addition of 0.5% protease inhibitor cocktail (Takara Bio.) was added and then homogenized in an ice bath using a Teflon–glass homogenizer (nine strokes, 800 rpm). After a centrifugation at 1,500 *g* for 5 min at 4°C, the supernatants were collected. To increase the yield, the pellet was washed twice with homogenization buffer and centrifuged at 1,500 *g* for 5 min at 4°C. The supernatants were combined and centrifuged at 18,000 *g* for 15 min at 4°C. The obtained pellets containing mitochondria were rapidly storedat −80°C before use for measurement of pyruvate dehydrogenase and α‐ketoglutarate dehydrogenase.

#### 2.4.5 ***ELISA for pyruvate dehydrogenase,*** *α* ***‐ketoglutarate dehydrogenase***

The concentration of pyruvate dehydrogenase 1, ketoglutarate dehydrogenase 1 and glucose‐6‐phosphate dehydrogenase in the mitochondrial fraction was measured using ELISA kits of rat pyruvate dehydrogenase 1 (ElAab Science co., Ltd., China), rat Ketoglutarate dehydrogenase 1 (ElAab Science co., Ltd., China) according to the manufacturer's instructions. Each sample was assayed in duplicate along with the kinase standards respectively. The absorbance at a wavelength of 450 nm was measured using iMark^TM^ Imcroplate Reader (Bio‐Rad). The concentration of the three enzymes was calculated from the standard curves and expressed as ng/mg protein.

#### 2.4.6 Determination of tissue ACh and AChE levels

The fresh PFC or hippocampus tissue (20 mg) was quickly removed from the brain and washed with wash buffer (20 mmol/L Tris‐HCl, pH 7.4). We homogenized each tissue sample in 500 ml of 0.9% saline containing 0.1 mol/L HClO_4_ in ice bath. After centrifugation at 1,500 *g* for 5 min at 4°C, the supernatants were collected. The supernatant (0.2 ml) was mixed with 0.3 ml distilled water and was followed by adding 0.05 ml 1.5 mmol/L of calabarine sulfate and 0.2 ml 2.0 mmol/L of trichloroacetic acid. The mixture was centrifuged at 5,000 *g* for 5 min. The collected supernatant (0.1 ml) was added to 0.1 ml of alkaline hydroxylamine hydrochloride (1.0 mol/L hydroxyl‐amine hydrochloride and 2.0 mol/L sodium hydroxide) and incubated at room temperature for 15 min. Then the mixture was reacted with 0.05 ml of 4.0 mol/L HCl and 0.05 ml of 0.4 mol/L ferric chloride (containing 0.1 mol/L HCl). Finally, 0.2 ml of each reaction mixture was spotted in duplicate onto 96‐well microplates followed by addition of 10 μl of 1.5 mmol/L physostigmine to inhibit the activity of acetylcholinesterase (AChE). After another 5 min of incubation at 37°C, the intensity of the brown ferric complex was read at 540 nm on an iMark^TM^ Imcroplate Reader (Bio‐Rad). Acetycholine (Ach) levels were expressed as μg/mg of tissue protein. AChE activity in the tissue homogenates was determined using a commercially available kit (Abnova). Briefly, the fresh tissue (25 mg) was quickly into 30 mmol/L sodium phosphate buffer (pH 7.0) and homogenized in ice bath; after centrifugation at 10,000 *g* for 5 min at 4°C, 5 μl of supernatant was added to a 96‐well plate and the volume was adjusted to 50 μl with AChE assay buffer; then 50 μl of reaction mix was added to each well containing choline standards, positive control and samples; after incubation for 30 min at 37°C, the absorbance at 570 nm was read on an iMark^TM^ Imcroplate Reader (Bio‐Rad). AChE activity was expressed as μg/mg of tissue protein.

### 2.5 Behavioral test

#### 2.5.1 Open field test

The open field apparatus was a square field (100 × 100 × 40 cm) made of black acrylic material. Rats of 90 days were placed in the corner of the apparatus and allowed to move freely for 10 min. The total distance and time spent in the center area (40 × 40 cm) were measured with the aid of a computer–based video tracking system. The data were analyzed using Image J OF4 (O'Hara & Co.), a modified software based on the public domain Image J Program.

#### 2.5.2 Morris water maze test

The water maze consisted of a circular pool (150 cm in diameter, 35 cm in wall height) with a transparent circular platform (12 cm in diameter) and was filled with water (25 ± 1°C), rendered opaque with white latex liquid. The platform was hidden 1 cm beneath water. The maze was sectioned into four equal quadrants: northwest (NW), northeast, southwest and southeast. The platform was placed at the NW quadrant. Differential visual spatial cues were placed on the cylindrical tank walls corresponding to the quadrant corners. The subjects were tested in a Morris water maze at the age of 90 days, and each animal was tested twice per day for five consecutive days. The rats were released into the pool from each of the two starting locations every day in a pattern that was randomly determined prior to testing. For every trial, the animal was placed in the pool facing the wall. Animals were then allowed 120 s to find the platform. If they were unable to find the platform in that time, they were guided to in by hand. They were allowed to remain on the platform for 15 s. After every trial, any visible feces were removed from the pool, and a minimum of 5 min elapsed between trials. All tests were initiated at noon, and the order in which the animals were tested was randomly changed. On the sixth day, the retention test was performed: the platform was removed, and the animal swam freely for 60 s. The time spent in the platform quadrant was measured as an index of memory retention. The path length and velocity were also recorded. Data were obtained with a tracking video system (Actimetrics).

#### 2.5.3 Three–chambered social behavioral test

This test was conducted to test the normal preference of a rat with a novel strange mouse, as opposed to a novel object. in a perspex arena (120 × 45 × 40 cm) that contained two tall, transparent Perspex boxes (20 × 22.5 × 40 cm) located in the opposite sides of the arena along the long dimension. The arena was visually divided into three areas (middle section: 20 × 45 × 40 cm; two end section: 50 × 46 × 40 cm), and opaque perspex sheets could be set in as closeable gates between the middle section and two end sections; transitions between the chambers and time spent in each chamber of the rats may be recorded by a computerized monitoring program. The rat subjected was placed in the closed middle chamber and allowed to acclimate for 10 min. Then a novel object (one of the four items that was approximately 10 cm tall and made of plastic) was placed into one of the boxes, and an unfamiliar rat was placed into the other box immediately prior to the test. The behavior of the rat was recorded for 10 min. The stranger rats had been previously acclimated to the boxes. The apparatus was cleaned and wiped with 70% ethanol at the onset of the investigations and between rats thereafter.

### 2.6 Statistical analysis

We used more than one pup from each litter in this research, which has been shown to inflate the probability of a Type 1 error (Maurissen, 2010), so we used an average value for each litter in the statistical analysis. All results are presented as means ± *SEM*. And all data were analyzed using the STATVIEW 5.0J software for Windows (SAS Inc). Student's *t*‐test was used to identify the difference between the control and BPA–treated groups. Statistical differences were considered significant when the *p*‐value was below 0.05.

## 3 RESULTS

The food intake, water consumption of the dams and the body weight of both dams and pups were monitored during the BPA treatment period. No difference was found between the BPA–exposed and control groups in any index. Maternal behaviors of the dams were also assessed and the results showed that BPA exposure did not affect any one dimension of the maternal behaviors observed including nursing, nest building, resting alone, grooming, active and eating/drinking (Data S1).

### 3.1 Serum total T_4_, total T_3_, free T_4_ and free T_3_ levels in dams and pups

**Figure 2:**
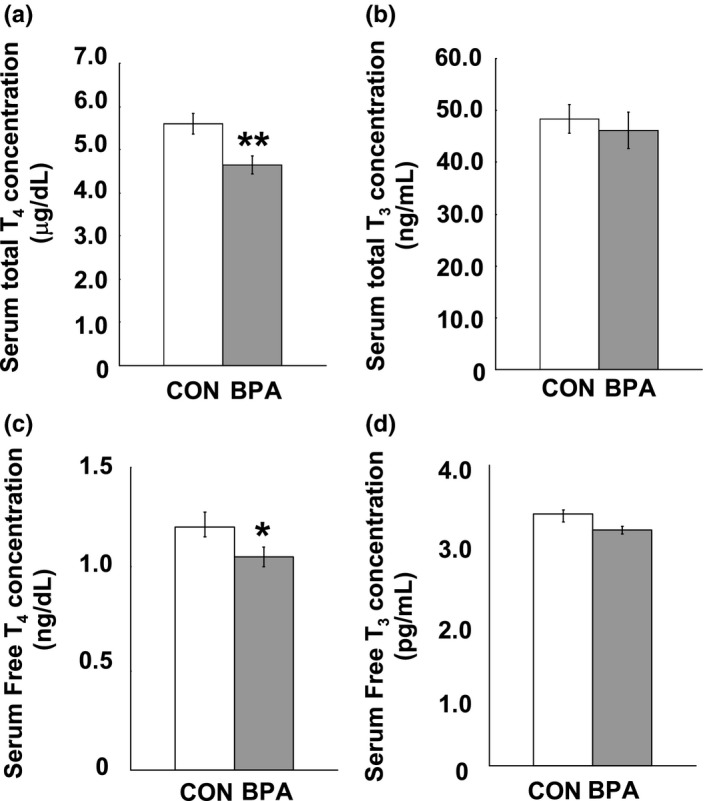
Serum concentration of thyroid hormones of dams measured at G21. (a) serum total T_4_ concentration; (b) serum total T_3_ concentration; (c) serum free T_4_ concentration; and (d) serum free T_3_ concentration. *N* = 10/group. Solid bars represent mean (±*SEM*). CON and BPA represent the control group and BPA–treated group, respectively. *p < 0.05, **p < 0.01, versus control group. BPA: bisphenol A; T_3_: triiodothyronine; T_4_: thyroxine

**Figure 3:**
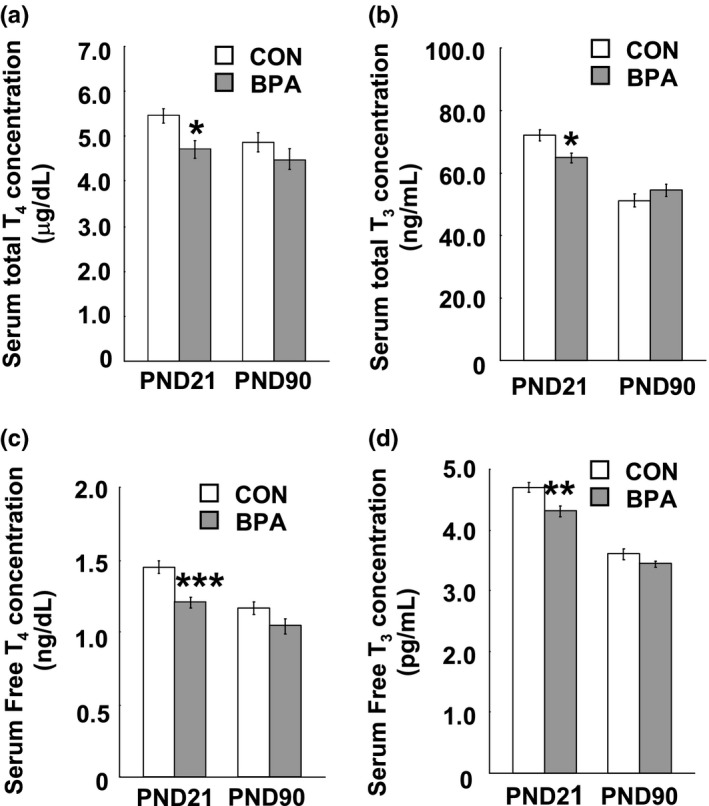
Serum concentration of thyroid hormones of male pups at PND21 and PND90. (a) serum total T_4_ concentration; (b) serum total T_3_ concentration; (c) serum free T_4_ concentration; and (d) serum free T_3_ concentration. *N* = 10/group/age. Solid bars represent mean (±*SEM*). CON and BPA represent the control group and BPA–treated group, respectively. *p < 0.05, **p < 0.01, ***p < 0.001 versus control group. BPA: bisphenol A; PND21: postnatal day 21; PND90: postnatal day 90; T_3_: triiodothyronine; T_4_: thyroxine

We monitored the serum total THs and free THs of both dams and pups. After 10 days of BPA exposure, significant decreases in total T_4_ (*p* < 0.01) and free T_4_ (*p* < 0.05) were observed in the serum of the BPA–exposed dams compared to control ones at G21 (Figure 2a,c), however, there was no significant difference in total T_3_ and free T_3_ between the control and BPA groups (Figure 2b,d). In the male pups, significant decrease in total T_4_ (*p* < 0.05) (Figure 3a), total T_3_ (*p* < 0.05) (Figure 3b), free T_4_ (*p* < 0.001) (Figure 3c) and free T_3_ (*p* < 0.01) (Figure 3d) were observed at PND21, while no difference was observedin total T_4_, free T_4_, total T_3_ and free T_3_ between the control and BPA–exposed groups at PND90 (Figure 3a–d).

### 3.2 TH levels in the PFC and hippocampus

**Figure 4:**
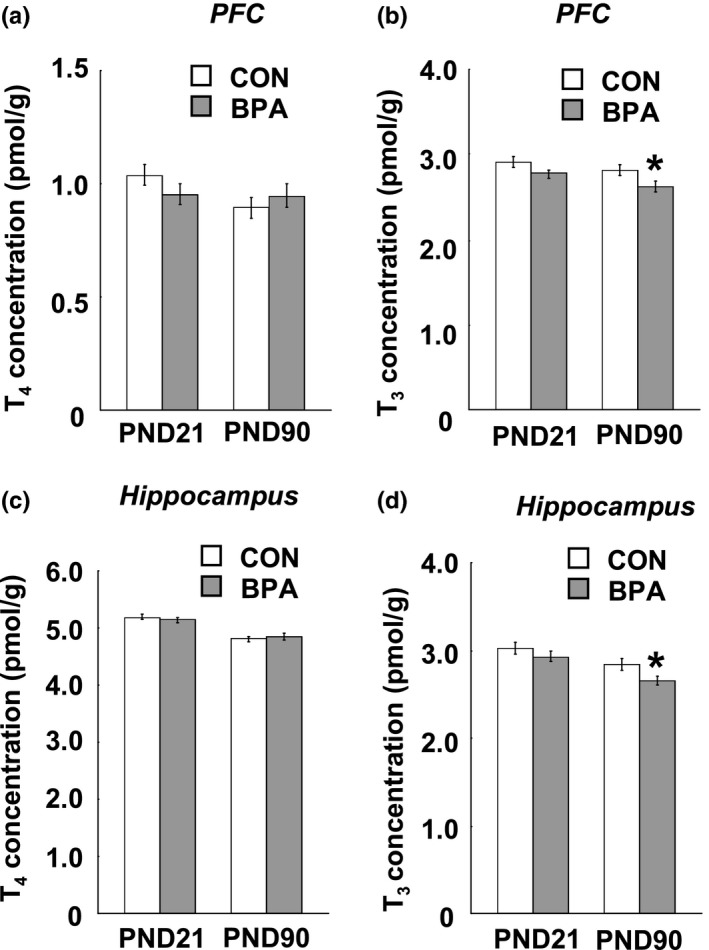
Tissue T_4_ and T_3_ levels in the PFC, hippocampus of the pups at PND21 and PND90. (a and b) Tissue T_4_ and T_3_ in the PFC respectively; (c and d) Tissue T_4_ and T_3_ in the hippocampus respectively, *n* = 10/group/age. Solid bars represent mean (±*SEM*). CON and BPA represent the control group and BPA–treated group, respectively. *p < 0.05 versus control group. BPA: bisphenol A; PFC: prefrontal cortex; PND21: postnatal day 21; PND90: postnatal day 90; T_3_: triiodothyronine; T_4_: thyroxine

Results of the T_4_ and T_3_ assay in the brain tissues of the pups are shown in Figure 4 In both the control and BPA–treated groups, a wide variability of T_4_ concentration was observed between the PFC (Figure 4a) and hippocampus (Figure 4c) at either PND21 or PND90, namely, the T_4_ concentration in the hippocampus was about fourfold higher than the one in the PFC within the duration examined (Figure 4a,c); conversely, the T_3_ levels in the two brain tissues were similar at PND21 and PND90 (Figure 4b,d). BPA exposure did not induce alteration of tissue T_4_ level in both the PFC and hippocampus at both PND21 and PND90 (Figure 4a,c), however, a significant decrease in tissue T_3_ concentration was observed in the PFC (*p* < 0.05) and hippocampus (*p* < 0.05) at PND21 but not PND90 (Figure 4b,d).

### 3.3 Expression of MCT8, OATP1c1, DIO2 and DIO3 mRNA in the PFC and hippocampus

**Figure 5:**
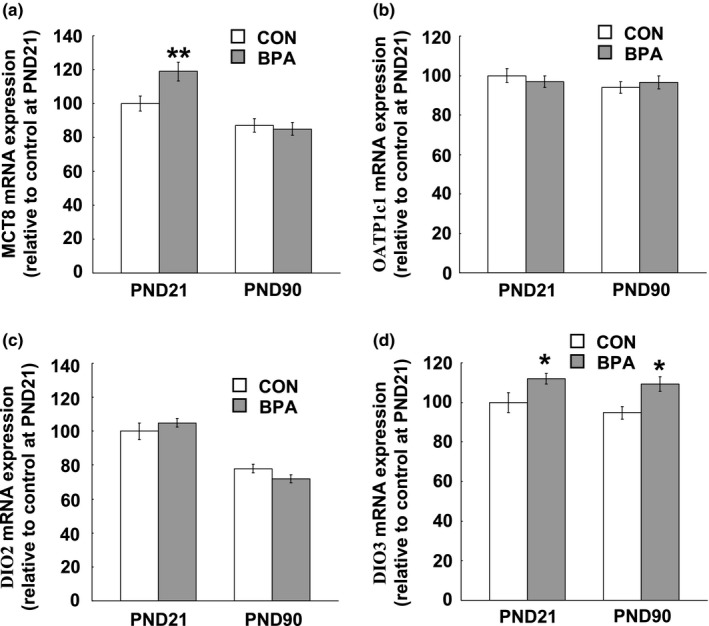
TH transporter and deiodinase mRNA expression levels in the PFC of the pups at age of PND21 and PND90. (a) MCT8 mRNA expression levels; (b) OATP1c1 mRNA expression levels; (c) DIO2 mRNA expression levels; (d) DIO3 mRNA expression levels. *N* = 10/group/age. Solid bars represent mean (±*SEM*). CON and BPA represent the control group and BPA–treated group, respectively. CON and BPA represent the control group and BPA–treated group, respectively. *p < 0.05, **p < 0.01 versus control group. BPA: bisphenol A; DIO: iodothyronine deiodinase; MCT8: monocarboxylate transporter 8; OATP1c1, organic anion–transporting polypeptide 1c1; PFC: prefrontal cortex; PND21: postnatal day 21; PND90: postnatal day 90; TH: thyroid hormone

**Figure 6:**
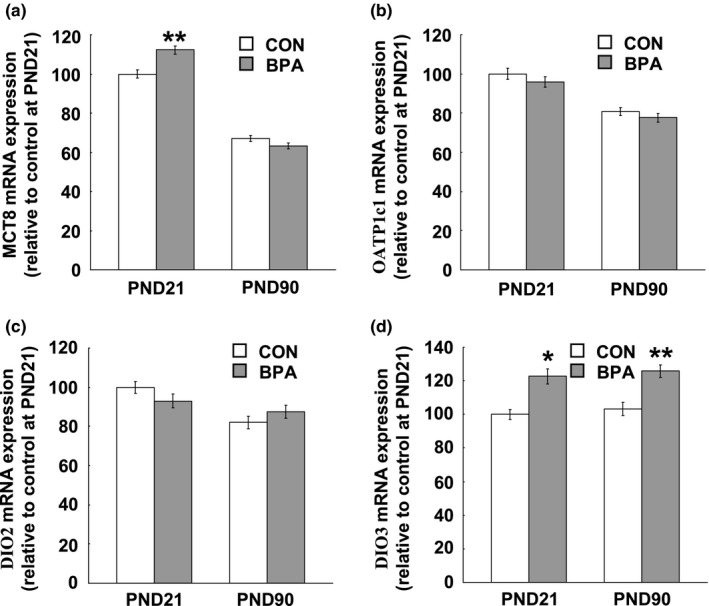
TH transporter and deiodinase mRNA expression levels in the hippocampus of the pups at PND21 and PND90. (a) MCT8 mRNA expression levels; (b) OATP1c1 mRNA expression levels; (c) DIO2 mRNA expression levels; (d) DIO3 mRNA expression levels. *N* = 10/group/age. Solid bars represent mean (±*SEM*). CON and BPA represent the control group and BPA–treated group, respectively. CON and BPA represent the control group and BPA–treated group, respectively. *p < 0.05, **p < 0.01 versus control group. BPA: bisphenol A; DIO: iodothyronine deiodinase; MCT8: monocarboxylate transporter 8; OATP1c1, organic anion–transporting polypeptide 1c1; PFC: prefrontal cortex; PND21: postnatal day 21; PND90: postnatal day 90; TH: thyroid hormone

The heterogeneous effects of BPA treatment on the serum and brain T_4_ concentrations strongly suggest the involvement of TH transporters localized in BBB. We examined the mRNA expression of MCT8 and OATP1c1 in the PFC and hippocampus at PND21 and PND90. The tissues from both hemispheres were used because no between–hemisphere difference was found in the mRNA expression of both TH transporters (data not shown). We observed a big expression variation in MCT8 and OATP1c1 mRNA between the PFC and hippocampus, namely, both the MCT8 and OATP1c1 mRNA expressions in the hippocampus were about threefold of one in the PFC at PND21 and PND90 (Figures 5a,b and 6a,b). Perinatal BPA exposure induced a remarkable increase in MCT8 mRNA expression in the PFC (*p* < 0.01, Figure 5a) and hippocampus (*p* < 0.01, Figure 6a) at PND21, while at PND90 no difference was seen in both the PFC and hippocampus between the control and BPA–treated groups (Figures 5a and 6a). Interestingly, BPA treatment did not affect OATP1c1 mRNA expression in both the two tissues at either PND21 or PND90 (Figures 5b and 6b).

The examination of DIO2 and DIO3 mRNA expression showed an opposing large variation in DIO2 and DIO3 expression levels between the PFC and hippocampus, namely, about twofold and threefoldhigher expression of DIO2 and DIO3 respectively, were observed in the PFC than the ones in the hippocampus at both PND21 and PND90 (Figures 5c,d and 6c,d). BPA treatment had no effect on DIO2 expression in both the PFC and hippocampus at both PND21 and PND90 (Figures 5c and 6c), while DIO3 mRNA expression in both the PFC and hippocampus at either PND21 (PFC: *p* < 0.05; Hippocampus: *p* < 0.05) or PND90 (PFC: *p* < 0.05; Hippocampus: *p* < 0.01) was significantly reduced by perinatal BPA exposure (Figures 5d and 6d).

### 3.4 Lactate and pyruvate concentrations in adult PFC and hippocampus

**Figure 7:**
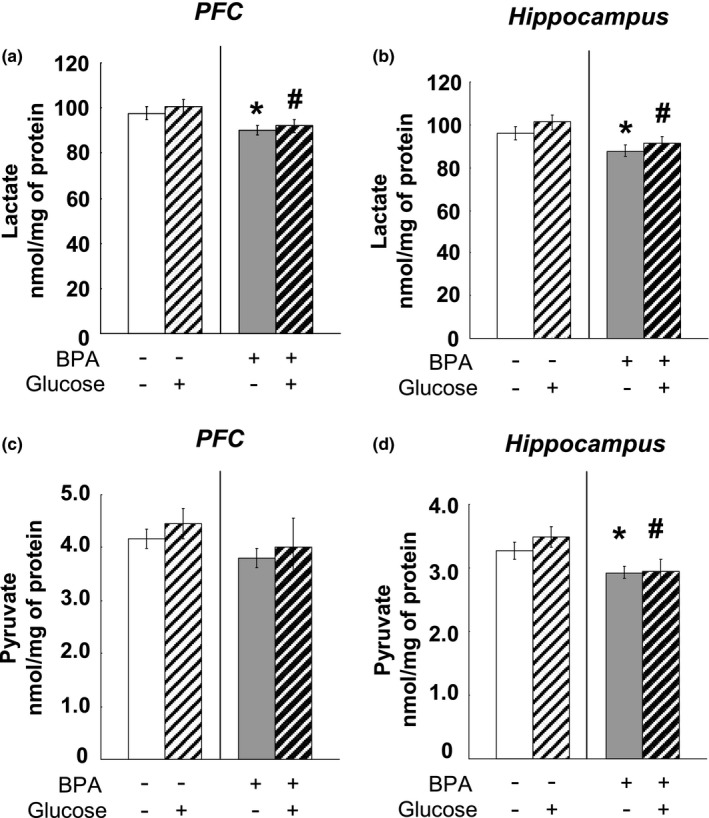
Effects of perinatal BPA exposure on the lactate concentrations in the PFC (a) and hippocampus (b) and pyruvate levels in the PFC (c) and hippocampus (d) at age of PND90, *n* = 10/group. Solid bars represent mean (±*SEM*). *p < 0.05 versus control group (without BPA treatment or glucose loading); ^#^ *p* < 0.05 versus the appropriate control group. BPA: bisphenol A; PFC: prefrontal cortex; PND90: postnatal day 90

As our above findings have illustrated that perinatal BPA treatment induced remarkable alteration of TH levels in matured PFC and hippocampus, the following question arises: whether the altered TH levels could be predictive of glucose metabolic changes in these brain tissues. As the main biochemical markers of glucose metabolism in the brain, pyruvate and lactate were measured in the PFC and hippocampus. Perinatal BPA exposure decreased lactate concentrations in adult PFC (*p* < 0.05) (Figure 7a) and hippocampus (*p* < 0.05) (Figure 7b). The pyruvate concentration in the hippocampus was significantly lower in animals subjected to BPA exposure (*p* < 0.05) (Figure 7d), however, BPA treatment did not alter the pyruvate levels in adult the PFC (Figure 7c).

### 3.5 The concentrations of key glycolytic enzymes and Krebs cycle enzymes in adult PFC and hippocampus

**Figure 8:**
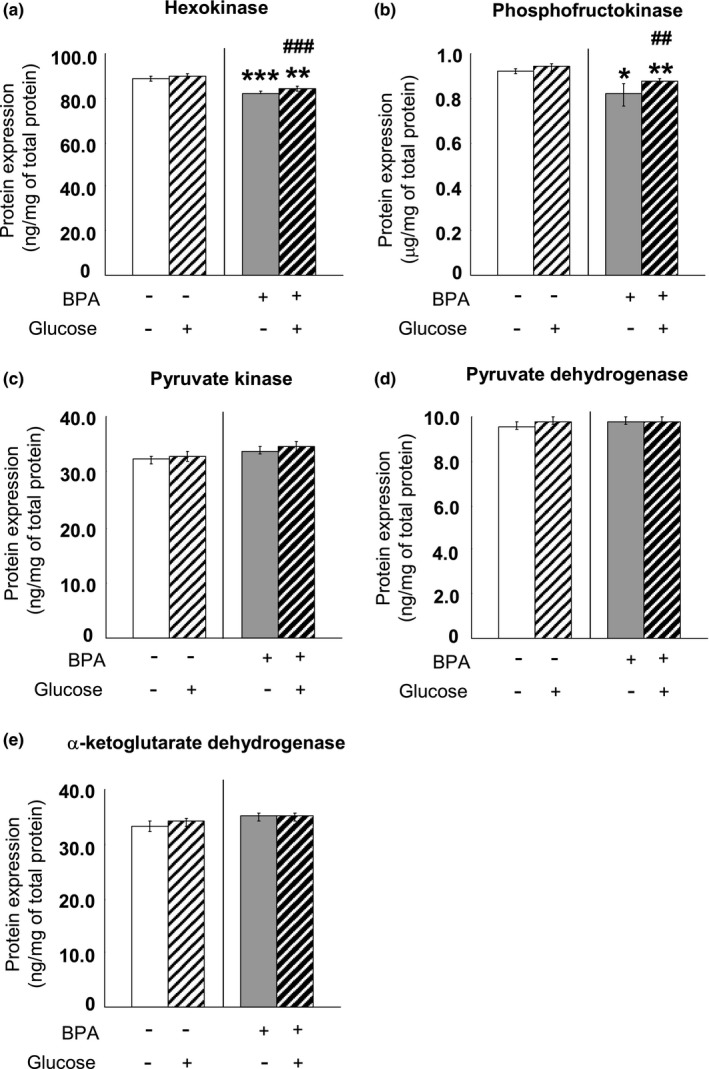
Effects of perinatal BPA exposure and glucose administration on hexokinase (a), phosphofructokinase (b), pyruvate kinase (c), pyruvate dehydrogenase (d) and α‐ketoglutarate dehydrogenase (e) in the PFC of the pups at age of PND90, *n* = 10/group. The results are expressed as mean (±*SEM*). *p < 0.05, **p < 0.01, ***p < 0.001 versus control group (without BPA treatment or glucose loading); ^##^ *p* < 0.01, ^###^ *p* < 0.001 versus the appropriate control group. BPA: bisphenol A; PFC: prefrontal cortex; PND90: postnatal day 90

**Figure 9:**
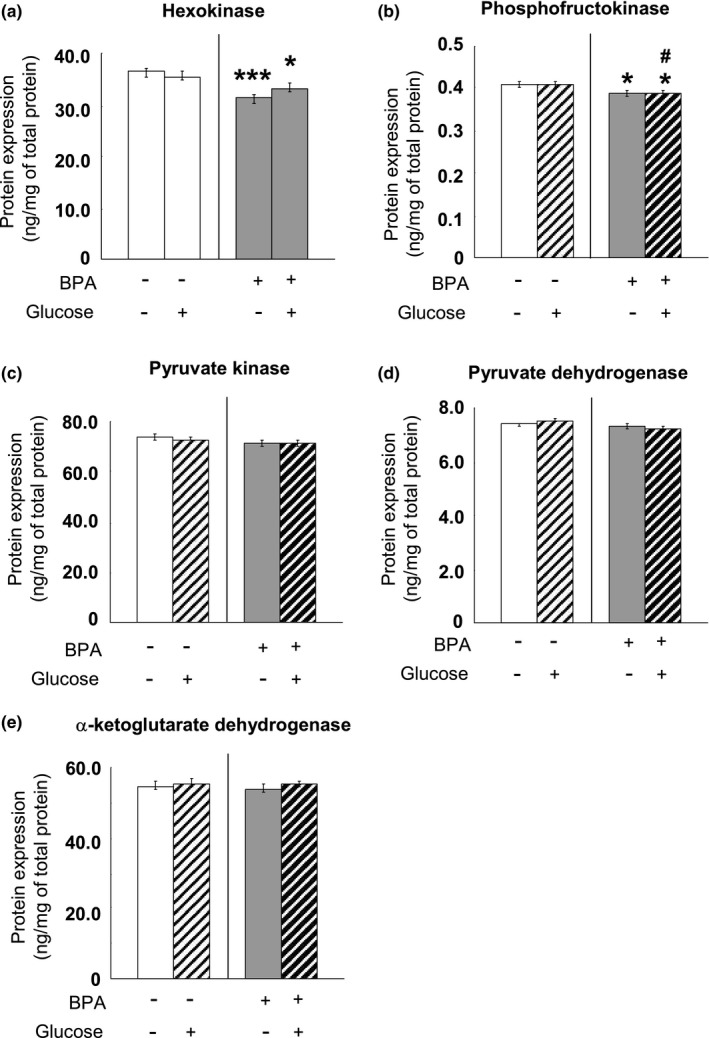
Effects of perinatal BPA exposure and glucose administration on hexokinase (a), phosphofructokinase (b), pyruvate kinase (c), pyruvate dehydrogenase (d) and α‐ketoglutarate dehydrogenase (e) in the hippocampus of the pups at age of PND90, *n* = 10/group. The results are expressed as mean (±*SEM*). *p < 0.05, ***p < 0.001 versus control group (without BPA treatment or glucose loading); ^#^ *p* < 0.05 versus the appropriate control group. BPA: bisphenol A; PND90: postnatal day 90

We examined the contents of three enzymes catalyzing the key, irreversible steps in the process of glycolysis, that is, hexokinase, phosphofructokinase and pyruvate kinase in the brain tissues at PND90. Moreover, as the most important enzyme responsible for irreversibly converting pyruvate to acetyl CoA and connecting glycolysis with the tricarboxylic acid cycle, pyruvate dehydrogenase 1 and α‐ketoglutarate dehydrogenase concentration was assessed in the PFC and hippocampus tissues. Perinatal BPA administration significantly decreased the Hexokinase and phosphofructokinase levels in the PFC (Hexokinase: *p* < 0.001; phosphofructokinase: *p* < 0.05) (Figure 8a,b) and hippocampus (Hexokinase: *p* < 0.001; phosphofructokinase: *p* < 0.05) (Figure 9a,b); however, BPA treatment did not affect the expression of pyruvate kinase (Figures 8c and 9c), pyruvate dehydrogenase (Figures 8d and 9d) and α‐ketoglutarate dehydrogenase (Figures 8e and 9e) mRNA in both the PFC and hippocampus. Glucose administration had no effect throughout all examination (Figures 8 and 9).

### 3.6 The ACh and AChE levels in adult PFC and hippocampus

**Figure 10:**
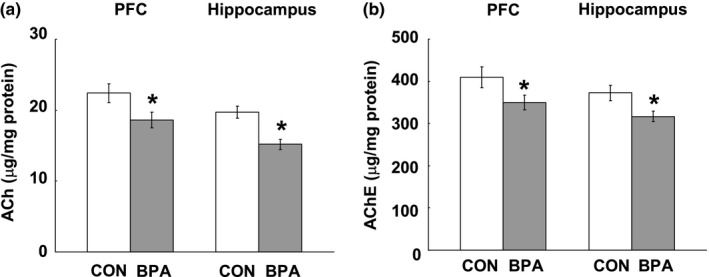
Effects of perinatal BPA exposure on ACh (a) and AChE (b) levels in the PFC and hippocampus at PND90, *n* = 10/group. The results are expressed as mean (±*SEM*). CON and BPA represent the control group and BPA–treated group, respectively. *p < 0.05, versus control group. Ach: acetycholine; AChE: acetylcholinesterase; BPA: bisphenol A; PFC: prefrontal cortex; PND90: postnatal day 90

Considering that the glucose metabolism has been reported to incorporate into Ach synthesis (Sims et al., 1980) and has a strong association with neuronal activity of cholinergic projection (Ouchi et al., 1996), we evaluated the effects of BPA exposure on Ach and AChE levels in the PFC and hippocampus of adult rats. Perinatal BPA exposure induced significant decrease in ACh level in the PFC (*p* < 0.05) and hippocampus (*p* < 0.05) at PND90 (Figure 10a), while decreased AChE level also was observed in both the PFC (*p* < 0.05) and hippocampus (*p* < 0.05) of BPA–exposed rats at PND90 (Figure 10b).

### 3.7 Locomotor activity, spatial memory and social behaviors

**Figure 11:**
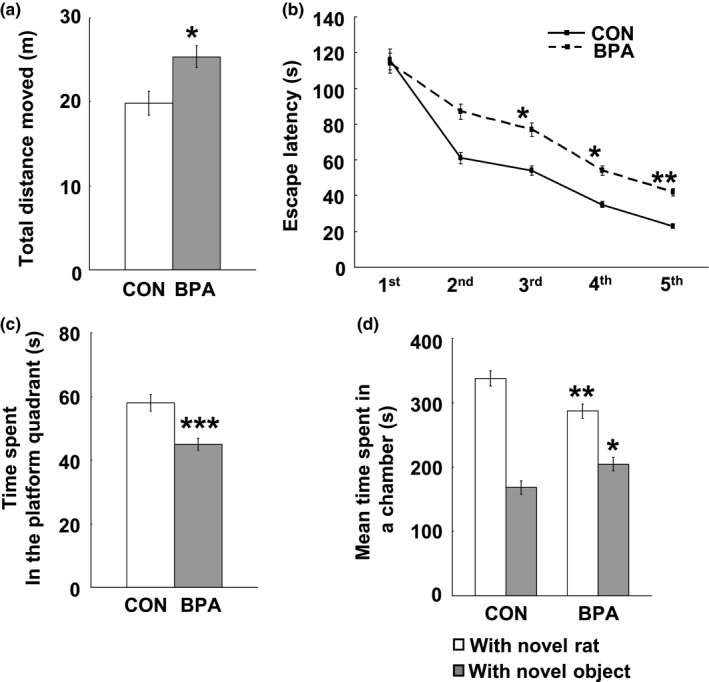
Effects of perinatal BPA exposure on locomotor activity (a), spatial learning (b), spatial memory (c) and social behavior (d) at PND90, *n* = 10/group. Solid bars represent mean (±*SEM*). CON and BPA represent the control group and BPA–treated group, respectively. *p < 0.05, **p < 0.01, ***p < 0.001 versus control group. BPA: bisphenol A; PND90: postnatal day 90

Our above findings have illustrated that perinatal BPA exposure induced a significant decrease in ACh and AChE levels in the PFC and hippocampus, we next examined whether the altered neurotransmitter level could be predictive of PFC/hippocampus–dependent behavioral changes. In the open field test, relative to the control rats, BPA–exposed rats moved a longer distance (*p* < 0.05, Figure 11a). In the Morris water maze task, the BPA–exposed rats took more time to find the target platform from the third day of training (3rd: *p* < 0.05; 4th: *p* < 0.05; 5th: *p* < 0.01, Figure 11b); while the subsequent probe test showed that BPA–exposed rats cruised less time in the platform quadrant than the controls (*p* < 0.001, Figure 11c). In the three–chambered social behavioral test, the BPA–exposed rats spent less time in both chambers with novel rat (*p* < 0.01) or novel object (*p* < 0.05) compared to control ones (Figure 11d).

## 4 DISCUSSION

In the present study, we found heterogeneous change in the TH concentrations between the serum and brain tissues, and alteration of MCT8 and DIO3 expressions in the PFC and hippocampus induced by BPA treatment. In addition, in the BPA–treated rats decreased glucose metabolism was found in the matured PFC and hippocampus (PND90) where decreased tissue T_3_ was observed, suggesting the long–term effects of low dose perinatal BPA exposure on brain TH homeostasis and glucose metabolism in adulthood.

Among the increasing researches showing strong relationship between BPA exposure and disturbance of TH homeostasis (Meeker & Ferguson, 2011; Wang et al., 2015), there was competing results of serum TH levels, decreased (Meeker & Ferguson, 2011; Wang et al., 2015; Xu et al., 2007) or increased (Delclos et al., 2014; Zoeller et al., 2005), induced by BPA exposure. The discrepancy may be caused by very different doses of BPA administration or administration methods. Previous studies have reported that the dose response of BPA was inverted U‐shaped which was also well observed in the biological effects of other hormones (Liang, Gao, Chen, Hong, & Wang, 2014; vom Saal et al., 1997), while gavage administration，used in a previous study reporting increased serum T_4_ after BPA treatment (Delclos et al., 2014) has been shown to result in aspiration, pulmonary injury, and/or elicitation of a stress response (Brown, Dinger, & Levine, 2000) which may consequently increase the levels of THs (Friedman, Bacchus, Raymond, Joffe, & Nobrega, 1999; Langer, Vigas, Kvetnanský, Földes, & Culman, 1983).

In mammals, the TH secretion and its concentration in blood is regulated by the hypothalamus‐pituitary‐thyroid axis, which controls the release of T_4_ by the thyroid follicles, while T_3_ activation and homeostasis is regulated by enzymatic deiodination activity in the peripheral tissues. Results of many authors suggested numerous mechanisms in the impairment of thyroid histo‐functional features by low dose of BPA exposure. The evidences in vitro include: (a) BPA impairs the thyrocytes transcriptome in a time–dependent manner (Porreca et al., 2016); (b) BPA inhibits iodide uptake and alters the transcriptional expression of three TH synthesis–related genes involved in Slc5as, Tpo, and Tgo and three thyroid transcription factor genes, Pax8, Foxe1 and Nkx2‐1 (Lee, Kim, Youn, & Choi, 2017; Wu, Beland, & Fang, 2016) and 3) BPA alters the size of the thyroid follicles with vacuolated colloids and thickening of the para follicular cells (Hernandez‐Rodriguez et al., 2007). Meantime, in vivo and clinical researches showed pathological effects of BPA on the thyroid gland including impaired thyroid volume (Wang et al., 2015) which is usually measured to evaluate the goiter status and then to assess the degree of iodine deficiency in a population, and increased risk of euthyroid antoimmune thyroiditis (Ademoglu, Keskin, Gorar, & Carlioglu, 2016). These evidences help us understand the changes in serum TH concentrations in BPA–treated rats, however, our finding that 70‐days (from PND21 to PND90) removal of BPA could recover the serum TH levels suggests that the effects of perinatal BPA treatment on TH synthesis may not endure when BPA was removed and metabolized.

T_4_ is thought to predominately enter the CNS in preference to T_3_ as a majority of BBB TH transporters exhibit greater affinities for T_4_ transport (Chatonnet, Picou, Fauquier, & Flamant, 2011; Heuer, 2007). After T_4_ is taken up into the brain, it is in turn converted locally to T_3_ by DIO2 followed by binding and activating TRs. The expression of TH transporters in a tissue–specific manner and the differed response of two the TH transporters are likely to contribute to the heterogeneous changes in T_4_ levels in the three brain tissues. Our qPCR analysis at PND21 showed a compensatory overexpression of MCT8 in the PFC and hippocampus to low serum TH levels supporting an important concept that the developing brain possesses potent mechanisms to compensate for small reductions in serum T_4_ and/or T_3_. To test this concept, Sharlin et al. have employed a model of grade serum T_4_ reductions using doses of propylthiouracil (PTU). They found that the DIO2 expression in the hippocampus was not affected by any grade of low serum T_4_, but RC3/neurogranin, a direct target of T_3_ action exhibited a strong negative linear correlation with serum total T_4_. In addition, their single–cell analysis of RC3 mRNA levels in cortical neurons demonstrated that the coexpression of MCT8 did not alter the relationship between RC3 and serum T_4_ (Sharlin, Gilbert, Taylor, Ferguson, & Zoeller, 2010). We found in their work, that the lowest dose of PTU almost induced a 35% reduction in serum T4 which is 2.4‐fold of the 14% reduction in serum T_4_ in the present study, which thus may exceed the degree to which putative compensatory mechanisms can ameliorate the consequences of slight serum hypothyrodism. As T_4_ transporter is abundant in the BBB of rodents, our results did not show any alteration in OATP1c1 expression in response to the serum hypothyrodism induced by BPA treatment. Although the underpinning mechanisms are unclear, the relatively conservative response to serum hypothyrodism is helpful for the stable TH circumstance within brain.

The regional deiodinases are critical determinants of the local T_3_ pool and therefore modulate nuclear T_3_ concentration and TR saturation. In rat brain, the local DIO2 activity is responsible for over 80% of the local T_3_ production (Crantz, Silva, & Larsen, 1982). It is easily understood from the finding that DIO2 expression was not affected in the PFC and hippocampus exposed to BPA in the case of normalized substrate (T_4_) level by compensation of MCT8 expression in the brain structures. Meaningfully, in the BPA–treated rats, despite the normal T_3_ level in the PFC and hippocampus at PND21，the DIO2 expression was up‐regulated and the effects endured into PND90; tying with our results showing that the DIO3 expression is very constant from PND21 to PND90 in either control or BPA–treated rats, we hypothesize that the expression pattern of DIO3 may be most determined during the developmental stage and remain relatively stable thereafter.

THs regulate systemic glucose metabolism and also are involved in the regulation of the brain glucose metabolism (Jahagirdar & McNay, 2012). The part of the present study concerning brain glucose metabolism showed a decreased amount of two important glycolytic enzymes, Hexokinase and phosphofructokinase, and lower concentrations of one of the products of glycolysis, lactate, in the 90 ‐day old PFC and hippocampus of the BPA–exposed rats. It is worth mentioning that the decrease in these two important enzymes in glycolysis regulation occurred in these brain regions with decreased T_3_ levels, confirming the strong link between TH and brain glucose metabolism. Insulin resistance may lead to glucose metabolism dysregulation (Arrieta‐Cruz & Gutiérrez‐Juárez, 2016), additionally, thyroid disorder including both hyperthyroid and hypothyroid are associated with insulin resistance (Kapadia, Bhatt, & Shah, 2012), we may hypothesize that the potential insulin signaling pathway is involved in the actions of brain hypothyroidism on weakening glycolysis in BPA–treated animals. Another point that should be mentioned is that in the present study we tested the concentration, but not the activity of the key glycolytic enzymes in order to evaluate the long–term changes in protein levels resulting from abnormal gene expression.

Many reports have suggested that the end product of glycolysis in the brain is lactate rather than pyruvate (Bélanger, Allaman, & Magistretti, 2011; Schurr & Payne, 2007). Current evidence showed that both astrocyte–neuron and oligodendrocyte—axon lactate shuttles are important for the brain energy metabolism and support neuronal function and survival (Cater, Chandratheva, Benham, Morrison, & Sundstrom, 2003; Fünfschilling et al., 2012). Our previous work has found that early BPA exposure induced decreased expression of monocarboxylate transporter 1 (MCT1), a critical protein for transporting lactate from oligodendrocytes to myelinated axons, and more demyelinated axons in the adult hippocampus of BPA–treated rats (Xu et al., 2017). The decrease in lactate production (present study) and decreased MCT1 expression (previous study) in the adult hippocampus of BPA–exposed rats are likely to cause weakness of axonal energy metabolism and consequent demyelination of axons, which may be responsible for deficit of contextual fear learning in adult BPA–treated rats (Xu et al., 2017).

In addition, some researches have shown that the glucose metabolism incorporates into acetylcholine (Ach) synthesis (Sims et al., 1980) and has a strong association with neuronal activity of cholinergic projection (Ouchi et al., 1996); moreover, activation of the forebrain cholinergic system has been demonstrated in PFC/hippocampus–dependent behaviors such as locomotor activity, performance of spatial memory task and social behaviors (Pepeu & Giovannini, 2004). In the present study, the decreased ACh levels in the PFC and hippocampus of BPA–exposed rats were probably induced by decreased glucose metabolism and/or direct disruption of BPA on ACh systhesis but not overdegradation because AChE levels were also decreased in BPA–exposed rat brains, and the decreased ACh level may take into account the changed performance in the several behavioral paradigms.

The present study showed that the serum TH level is not an appropriate index for evaluating TH contents in the brain. Moreover, this study supported the concept that a developing brain possesses mechanisms to compensate for moderate serum hypothyrodism to match the demands of the brain for normal development. In the meantime, this study also noticed the long–term negative effects of moderate serum hypothyrodism induced by perinatal BPA exposure or not，which may threaten public mental health.

## CONFLICTS OF INTEREST

None declared.

## AUTHOR CONTRIBUTIONS

Xiaobin Xu, contributed to writing and editing. Shijun Fan, Yuanqiao Guo, contributed to data analysis and chart making. Ruei Tan, Junyu Zhang, Wenhua Zhang, Nobumasa Kato contributed to methodology. Xiaobin Xu, Bing‐Xing Pan, contributed to project administration.
