# Supplementary material for: The effects of perinatal bisphenol A exposure on thyroid hormone homeostasis and glucose metabolism in the prefrontal cortex and hippocampus of rats
Source: Brain Behav. 2019 Feb 13;9(3):e01225. doi: 10.1002/brb3.1225 (PMC6422808; doi:10.1002/brb3.1225)

**1. Daily food intake, water consumption and body weight of dams and pups**

The food intake, water consumption of the dams and the body weight of dams and pups were monitored daily throughout the examination. BPA treatment induced no difference in daily food intake and water consumption, meanwhile, the body weight of the dams (from G14 to L21) and male pups (from PND1 to PND90) was not affected by BPA exposure (Table 1).

**2. Maternal Behavior**

Maternal behaviors were assessed by observing lactating dams in their home cages during a period of 90 minutes on PNDs 2-21. In this experiment the observation period started at 0930 hr and was conducted entirely during the dark phase with the aid of 20-W red lights. Each dam was observed once every 3 minutes for the following measures: a) nursing: the dam was allowing the pups (not necessarily the whole litter) to suckle，or that the dam was adopting the nursing posture with her body arched over the pups; b) nest building: the dam was engaged in some aspect of nest building either inside or outside the nest itself; c) eating/drinking: the dam was eating food pellet or drinking from the water bottle; d) grooming: the dam was grooming her own body; e) active: the dam was moving about the cage; f) resting: the dam was lying motionless outside the nest, not involved in any other behavior and with no pup attached to her nipples. The results showed no effects of BPA treatment on the dams’ maternal behaviors as below.


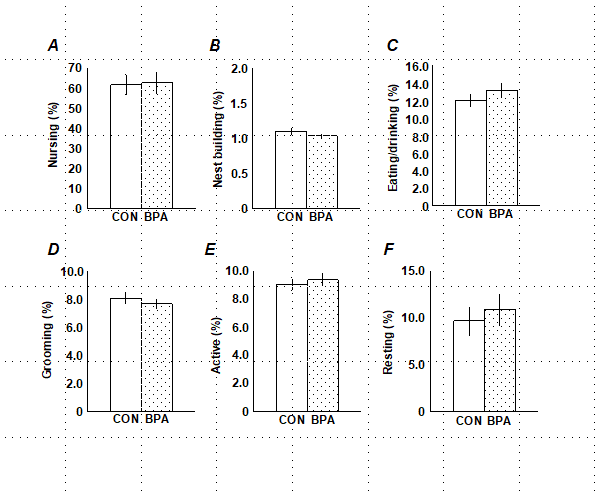

Supplement: Supplementary file 1 [file BRB3-9-e01225-s001.doc]
